# Transbilayer Dynamics of Phospholipids in the Plasma Membrane of the *Leishmania* Genus

**DOI:** 10.1371/journal.pone.0055604

**Published:** 2013-01-30

**Authors:** Marcos Gonzaga dos Santos, Sandra Marcia Muxel, Ricardo Andrade Zampieri, Thomas Günther Pomorski, Lucile Maria Floeter-Winter

**Affiliations:** 1 Departamento de Fisiologia, Instituto de Biociências, Universidade de São Paulo, São Paulo, Brazil; 2 Center for Membrane Pumps in Cells and Disease - PUMPKIN, Danish National Research Foundation, Department of Plant Biology and Biotechnology, University of Copenhagen, Frederiksberg, Denmark; Cornell University, United States of America

## Abstract

Protozoans of the *Leishmania* genus are the etiological agents of a wide spectrum of diseases commonly known as leishmaniases. Lipid organization of the plasma membrane of the parasite may mimic the lipid organization of mammalian apoptotic cells and play a role in phagocytosis and parasite survival in the mammal host. Here, we analyzed the phospholipid dynamics in the plasma membrane of both the *L. (Leishmania)* and the *L. (Viannia)* subgenera. We found that the activity and substrate specificity of the inward translocation machinery varied between *Leishmania* species. The differences in activity of inward phospholipid transport correlated with the different sensitivities of the various species towards the alkyl-phospholipid analogue miltefosine. Furthermore, all species exhibited a phospholipid scramblase activity in their plasma membranes upon stimulation with calcium ionophores. However, binding of annexin V to the parasite surface was only detected for a subpopulation of parasites during the stationary growth phase and only marginally enhanced by scramblase activation.

## Introduction

Protozoans of the genus *Leishmania* cause a complex disease called leishmaniases, whose clinical manifestations have been divided into three principal types that exhibit different degrees of severity and mortality: cutaneous, mucocutaneous, and visceral [1]. Species of this genus alternate between an intracellular amastigote stage in the mammalian host and a free, flagellated promastigote stage in the gut of the sand fly vector. Lainson and Shaw [2] have divided the genus *Leishmania* into two subgenera, *L. (Vianna)* and *L. (Leishmania)*, based on the developmental stage of the parasite in the gut of the sand fly vector. Members of the subgenus *L.* (*Leishmania),* including *L. (L.) amazonensis*, *L. (L.) donovani*, *L. (L.) infantum*, and *L. (L.) tropica*, develop exclusively in the midgut and foregut of the sand fly, whereas members of the subgenus *L. (Viannia)*, including *L.(V.) braziliensis* and *L. (V.) guyanensis*, have an additional development phase in the hindgut.

A critical point in the host-parasite interaction involves the attachment of the parasite to the intestinal epithelium and a posterior detachment and migration to the proboscis of the insect vector. In mammals, this host-parasite interaction involves the attachment to and invasion of host macrophages, initially by metacyclic promastigotes and later by amastigotes. Both metacyclic and amastigotes use receptor-mediated phagocytosis for mammalian cell invasion. The phospholipid phosphatidylserine (PS) has been suggested as a crucial ligand of the parasite in this process [3]–[5]. Although recent results suggest that *Leishmania* parasites lack PS [6]–[9], the detection of cell surface lipids by staining with labeled annexin V, a protein that interacts with PS but also with phosphatidic acid and phosphatidylinositol-4,5-bisphosphate [10], is consistent with the hypothesis that the protozoan parasite might take advantage of a regulated surface-display of specific lipids to invade and survive in host macrophages.

Typically, the eukaryotic plasma membrane displays an asymmetrical distribution of phospholipids across the bilayer, with PS and phosphatidylethanolamine (PE) restricted to the cytoplasmic leaflet and sphingolipids enriched in the exoplasmic leaflet. This distribution is maintained by energy-dependent inward translocation enzymes called flippases that use ATP hydrolysis to translocate specific lipids against a concentration gradient across the bilayer [11], [12]. In addition to these energy-dependent flippases, certain eukaryotic cells contain a phospholipid scramblase: a putative membrane protein that upon activation facilitates a rapid bidirectional movement of phospholipids across the two plasma membrane leaflets, disrupting the lipid asymmetry created by the ATP-dependent flippases [13]–[15].

Not much is known about transbilayer dynamics of phospholipids and the involvement of proteins in the plasma membrane of *Leishmania*. Previous studies performed with *L. (L.) infantum*, *L. (L.) donovani* and *L. (L.) tropica* have shown an ATP-dependent, inward-directed flippase activity [16]–[18]. This flippase activity requires at least two plasma membrane proteins: LdMT, a member of the P4 subfamily of P-type ATPases involved in phospholipid translocation, and LdRos3 (the β subunit of LdMT), a member related to the Cdc50 family [19], [20]. Studies on *L. (L.) tropica* and *L. (L.) infantum* have linked ATP-binding cassette transporters to active trafficking and outward transport of phospholipids across the parasite plasma membrane [21]–[24]. Furthermore, stimulation of *L. (L.) donovani* with calcium ionophores has recently been shown to trigger a phospholipid scramblase activity that facilitates bidirectional movement of phospholipids independent of cellular ATP [20].

Despite the clinical relevance of species of the *L. (Viannia)* subgenus, there have been few reports on their lipid organization. In this work, we compared phospholipid dynamics in the plasma membrane of the *L. (Leishmania)* and the *L. (Viannia)* subgenera. First, we studied the redistribution of fluorescent phospholipid analogues by flow cytometry. We found that the activity and substrate specificity of the inward translocation machinery varies between *Leishmania* species and that all species exhibit a phospholipid scramblase activity upon stimulation with calcium ionophore. Furthermore, all parasite species could be stained by FITC-labeled annexin V (annexin V-FITC) upon digitonin-permeabilization but binding of annexin V-FITC to the parasite surface was only detected for a subpopulation of parasites during the stationary growth phase and only marginally enhanced by scramblase activation.

## Results

### Phospholipid transport activity in the plasma membrane of *Leishmania* parasites

**Figure 1:**
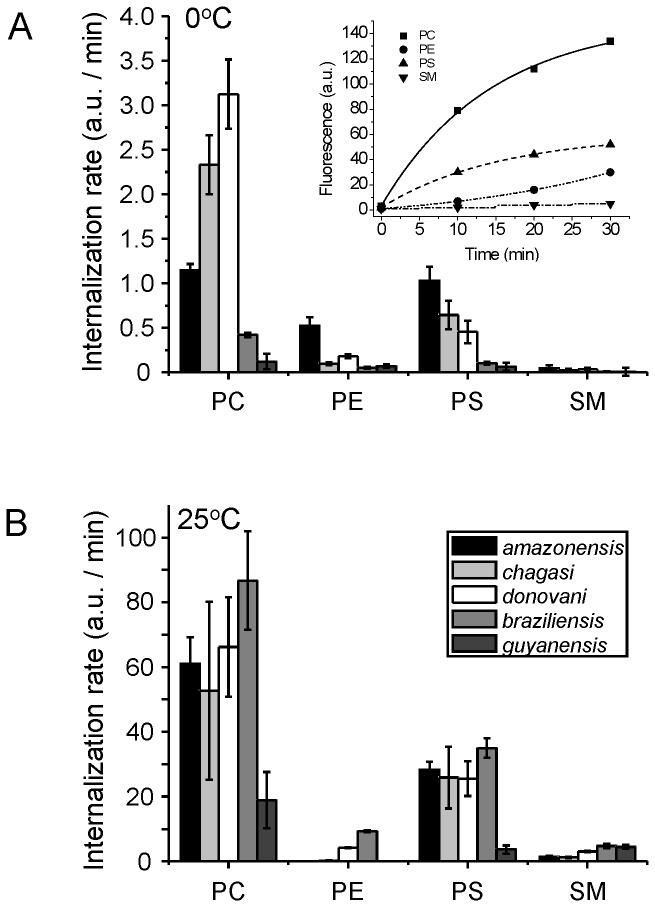
Internalization of NBD-labeled phospholipids from the plasma membrane of various *Leishmania* species. The internalization of NBD-PC, NBD-PE, NBD-PS and NBD-SM in cultured log-phase promastigotes at 2°C (A) and 25°C (B) were analyzed by flow cytometry using back-exchange to albumin. The internalization rates are calculated by fitting the data to a single-exponential equation and are shown as column charts reflecting the mean ± SD of three independent experiments. *Inset*: Time course of NBD-lipid internalization in *L. (L.) amazonensis*. Plotted lines represent the best fit to a single-exponential curve determined by the least-square method.

To compare the activity and characteristics of lipid transporters in the plasma membrane of various *Leishmania* species promastigotes, we first examined the amount of inwardly transported 7-nitrobenz-2-oxa-1,3-diazole (NBD)-labeled phospholipids, which is protected against albumin extraction after the translocation, by flow cytometry. Experiments were performed at 2°C to suppress endocytosis [16]. Under these conditions, all studied species displayed pronounced differences in the internalization of NBD-phosphatidylcholine (NBD-PC), NBD-PS and NBD-PE, but did not internalize NBD-sphingomyelin (NBD-SM) (Fig. 1A).

In combination with fluorescence microscopy, flow cytometry and chemical analysis we previously demonstrated that the increase of the cell-associated NBD fluorescence over time results from the transport of intact NBD-lipids to the cell interior [18], [25]. For *L. (L.) amazonensis, L. (L.) chagasi*, and *L. (L.) donovani*, rapid and selective internalization of NBD-PC and NBD-PS were observed with transport rates at least 24- and 12-fold higher, respectively, than NBD-SM (Fig. 1A). This rapid and selective internalization was severely inhibited in ATP-depleted parasites (Table 1), although the inhibition was not as pronounced in *L. (V.) braziliensis* and *L. (V.) guyanensis* (Table 1). All studied species except *L. (L.) amazonensis* did not significantly internalize NBD-PE (Fig. 1A).

**Table 1:** Effect of the depletion of intracellular ATP on NBD-PC internalization.^a^

	Internalization rate (a.u./min)		
	Control	- ATP^b^	% of Control^c^
*L. (L.) amazonensis*	4.87±0.66	0.91±0.01	18.8±2.0
*L. (L.) chagasi*	2.48±0.10	0.64±0.18	25.9±7.3
*L. (L.) donovani*	2.04±0.14	0.20±0.13	9.7±6.3
*L. (V.) braziliensis*	4.40±0.20	2.81±0.27	63.9±6.1
*L. (V.) guyanensis*	3.33±0.29	2.12±0.43	64.4±18.5

a Internalization of NBD-PC in cultured log-phase promastigotes at 2°C was analyzed by flow cytometry using back-exchange to BSA. The internalization rates were calculated by fitting the data to a single-exponential equation as described in Materials and Methods.

b ATP depletion was performed by pretreating parasites with 5 mM 2-deoxyglucose and 5 µg/mL oligomycin for 30 min at 25°C.

c Results are expressed relative to the NBD-PC internalization rate of untreated cells and are shown as the mean ± SD of three independent experiments.

For *L. (L.) amazonensis, L. (L.) chagasi*, and *L. (L.) donovani*, a striking difference in the internalization rates between the four NBD-lipids was also found when the incubation was performed at 25°C (Fig. 1B). *L. (V.) braziliensis* displayed a similar rapid and selective internalization of NBD-PC and NBD-PS under these conditions. *L. (V.) guyanensis* showed even lower NBD-PC and NBD-PS internalization rates compared to the other tested species, with transport rates similar to that of NBD-SM. These findings correlated with the different sensitivities of the different species towards miltefosine, an alkyl phospholipid analog of PC. *L. (L.) amazonensis, L. (L.) chagasi* and *L. (L.) donovani* were at least 1.7-fold more sensitive to miltefosine compared to *L. (V.) braziliensis* and *L. (V.) guyanensis* (Table 2). Collectively, these data are consistent with the presence of an active inward transport activity in the plasma membrane of both *Leishmania* subgenera; however, the activity and specificity varies between species.

**Table 2:** *Leishmania* miltefosine sensitivity.^a^

	EC_50_
L. (L.) amazonensis	7.96±0.17
L. (L.) chagasi	1.86±0.16
L. (L.) donovani	1.31±0.08
L. (V.) braziliensis	23.88±0.44
L. (V.) guyanensis	13.65±0.94

a Cultured mid-log-phase promastigotes were seeded at 5×10^5^ cells mL^−1^ with different miltefosine concentrations (5–200 µM), and the cell density was determined after 48 h. The EC_50_ values are shown as the mean of miltefosine concentration (µM) ± SD of two independent experiments.

### Phospholipid scramblase activity in the plasma membrane of ionomycin-treated parasites

**Figure 2:**
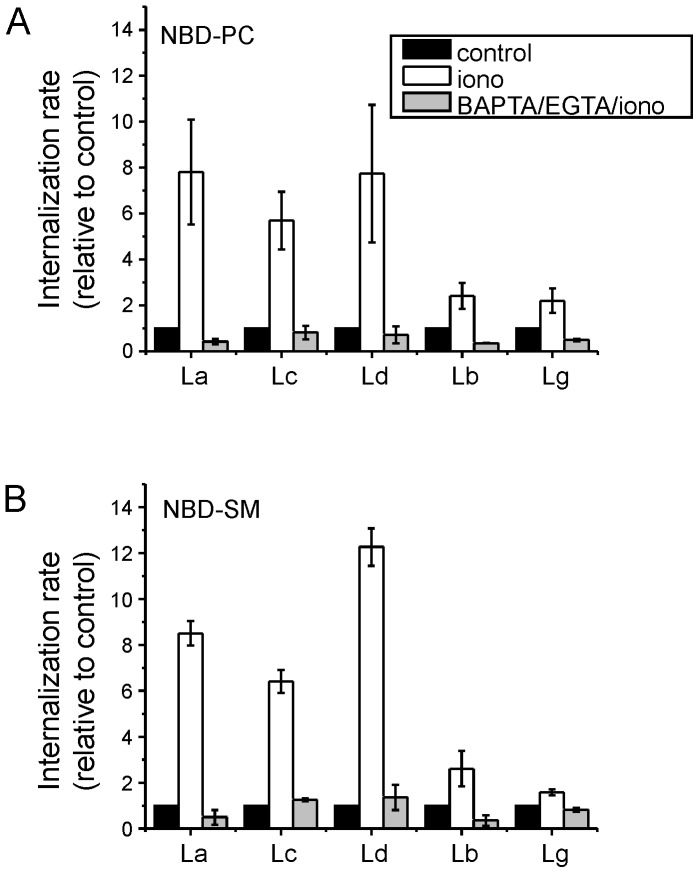
Internalization of NBD-labeled phospholipids in calcium ionophore-treated *Leishmania*. Log-phase promastigotes were left untreated (control) or pretreated for 15 min with 40 µM ionomycin (iono). For calcium depletion, BAPTA-AM and EGTA were added 30 min before addition of ionomycin (BAPTA/EGTA/iono). Subsequently, the internalization of NBD-PS (A) and NBD-SM (B) in parasites at 2°C was analyzed by flow cytometry using back-exchange to albumin. The internalization rates were calculated by fitting the data to a single-exponential equation. All data was normalized to the NBD-phospholipid internalization of untreated cells (control) defined as 1, and are shown as in column charts as the mean ± SD of two independent experiments. La, *L. (L.) amazonensis*; Lb, *L. (V.) braziliensis*; Lc, *L. (L.) chagasi*; Ld, *L. (L.) donovani*; Lg, *L. (V.) guyanensis*.

We recently provided evidence for a Ca^2+^-induced phospholipid scrambling activity in the plasma membrane of *L. (L.) donovani* during ionomycin or thapsigargin stimulation [20]. To elucidate whether this activity is a general feature of *Leishmania* parasites, we next analyzed the internalization of NBD-labeled phospholipids in the plasma membrane of the five studied species upon ionomycin stimulation (Fig. 2). NBD-PS was selected for this experiment because of potential role of PS in infection [3], [5], [26]. NBD-SM was selected as an indicator of nonspecific transport.

To make it easier to visualize the effect, we considered the transport rate of the non-treated control cells as 1, for both NBD-lipids. For all five species, stimulation with ionomycin (in a Ca^2+^-containing medium) enhanced the internalization rate of both NBD-lipids, resulting in transport rates at least 1.6-fold higher than the transport rates of non-treated cells (Fig. 2). For the two *L. (Viannia)* species, the observed increase in the NBD-lipid internalization rate was not as high as that observed for the *L. (Leishmania)* species (1.6 to 2.4-fold and 5.7 to 12.3-fold for *L. (Viannia)* and *L. (Leishmania)*, respectively), but the rate was significantly greater than the rate observed with the untreated controls (P<0.05). In the presence of EGTA (a chelator of extracellular calcium) and BAPTA-AM (a chelator of intracellular calcium), ionomycin treatment had no effect on lipid internalization, indicating that the stimulation is Ca^2+^-dependent and not a non-specific effect of ionomycin addition (Fig. 2). Extraction and chromatographic analysis of the fluorescent lipids after 45 min of incubation at 2°C did not reveal altered lipid metabolism in the presence of ionomycin. Less than 20% of the probes were converted to other NBD-lipid derivatives, irrespective of the parasite species (data not shown). Collectively, these findings are consistent with Ca^2+^-induced phospholipid scrambling in the plasma membrane of all five *Leishmania* species.

### A *Leishmania* subpopulation binds annexin V upon entering stationary-phase

**Figure 3:**
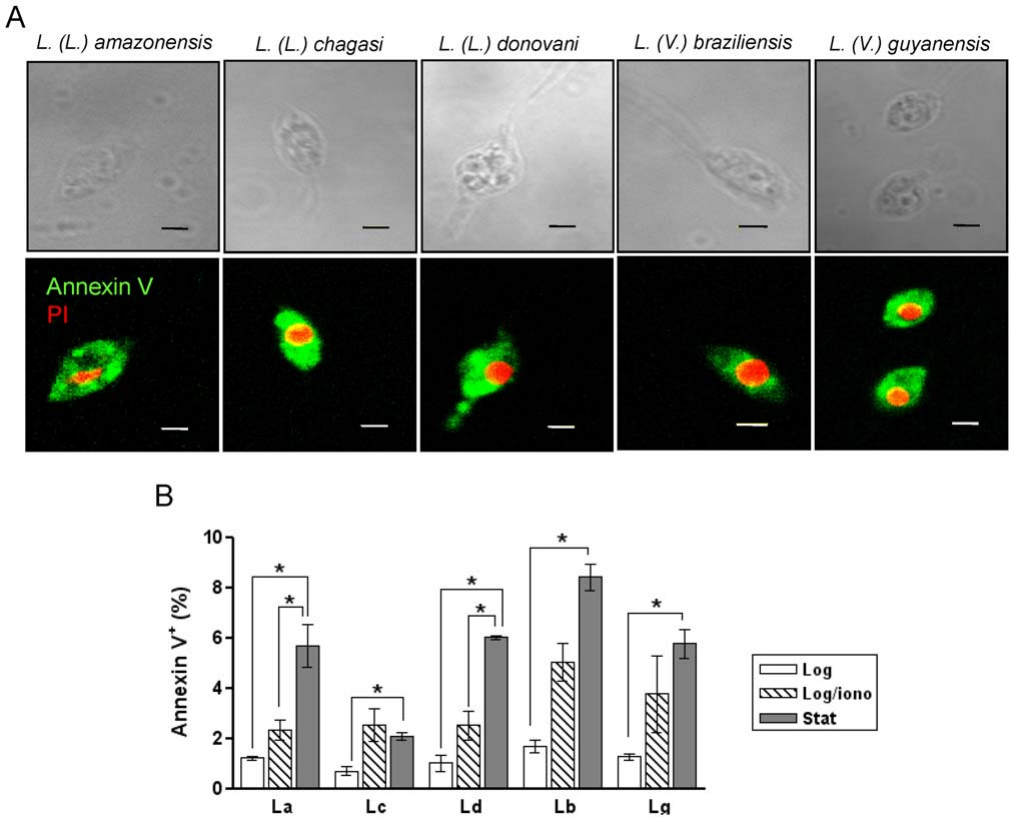
Annexin V-FITC binding in *Leishmania* species. (A) Promastigotes permeabilized with digitonin were incubated with annexin V-FITC and propidium iodide (PI) for 20 min at 2°C. Cells were transferred onto poly-L-lysin–coated slides and immediately analyzed by phase contrast and confocal fluorescence microscopy. Green, annexin V-FITC; Red, PI. The bars indicate 5 µm. (B) Log-phase (Log, white bars) and stationary-phase (Stat, gray bars) promastigotes were stained with annexin V-FITC and PI followed by flow cytometry analysis. Alternatively, log-phase promastigotes were incubated for 20 min with annexin V-FITC and PI in the presence of 40 µM ionomycin (Log/iono, stripped bars) and then analyzed by flow cytometry. The PI positive events were gated out of the analysis (right quadrants, Supplemental Fig. S1) and the fraction of the annexin V-positive cells was calculated as a percentage in the PI negative population. Results represent the mean ± SD of two independent experiments. Significant differences were determined by Student paired t-tests and denoted by asterisks (P<0.05). La, *L. (L.) amazonensis*; Lc, *L. (L.) chagasi*; Ld, *L. (L.) donovani*; Lb, *L. (V.) braziliensis* (Lb); and Lg, *L. (V.) guyanensis*.

Next, we investigated the staining of log- and stationary-phase promastigotes with annexin V-FITC, a lipid-binding protein that preferentially interacts with membranes containing anionic phospholipids and that is typically used to detect PS displayed on the cell surface. For all studied species, digitonin permeabilization resulted in strong FITC labeling of the parasites, indicating the presence of at least one class of lipids with annexin V-binding capability (Fig. 3A). Quantitative analysis of propidium iodide (PI)-negative promastigotes by flow cytometry (Fig. S1) showed that log-phase parasites hardly exhibited annexin V-binding at their cell surface (Fig. 3B), indicating that under these conditions lipids with annexin V-binding capability are confined to the inner plasma membrane leaflet or intracellular membranes. Upon entering the stationary growth phase, a subpopulation of PI-negative promastigotes bound annexin V on its surface (Fig. S1), indicating a shift in the phospholipid distribution in the plasma membrane. This feature was observed for *L. (L.) amazonensis*, consistent with data previously reported [5], and also for all other tested species (Fig. 3B, compare white and gray bars). Notably, stimulation of log-phase promastigotes by ionomycin resulted only in a marginal increase in the annexin V-positive subpopulation as compared to non-treated, stationary phase cells, except for *L. (L.) chagasi* (Fig. 3B, compare white and striped bars). This result is in sharp contrast with the lipid analog translocation upon ionomycin stimulation, where >90% of the cell population displayed an increase in lipid analog translocation rates (data not shown).

**Figure 4:**
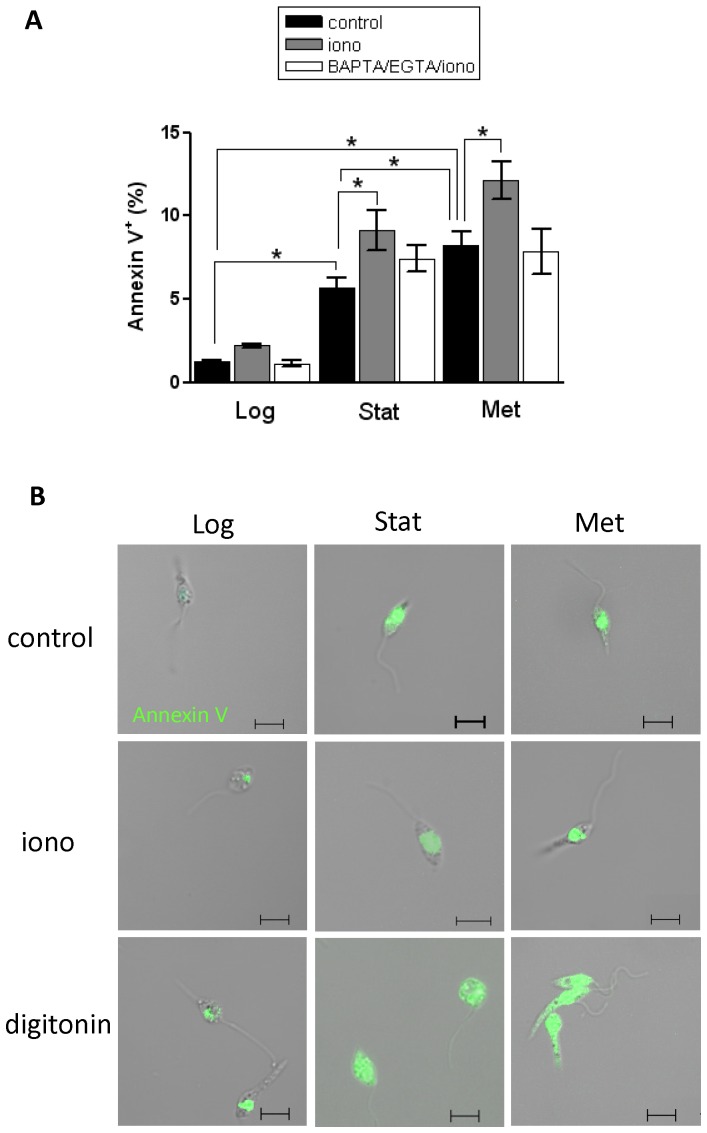
- Annexin V-FITC binding in *L. (L.) amazonensis*. (A) Bar diagram of the percentage of annexin V-FITC positive population calculated as explained in fig 3. Log-phase (Log), stationary-phase (Stat) and metacyclics-enriched (Met) promastigotes left untreated (control) or treated with 40 µM ionomycin (iono) were labeled for 20 min at 2°C with annexin V-FITC and PI, and subsequently analyzed by flow cytometry or microscopy. For calcium depletion, BAPTA-AM and EGTA were added for 30 min at 2°C before adding ionomycin (BAPTA/EGTA/iono). Results represent the mean ± SD of two independent experiments. Significant differences were determined by Student paired t-tests and denoted by asterisks (P<0.05). (B) Annexin V-FITC staining (green) combined with phase-contrast micrographs are depicted. Control and ionomycin treatments were as described in A. The bars indicate 5 µm.

Finally, we assessed the annexin V-binding to *L. (L.) amazonensis* parasites harvested in log-phase and in stationary phase as well as to a metacyclics enriched population (Fig. 4). In line with a previous report [5], the metacyclics enriched population displayed a higher frequency of annexin V-positive cells with 8.2±2.5% (n = 6) compared to 5.7±2% (n = 6) in the total population of stationary-phase parasites. Again, in all cases stimulation of the parasites by ionomycin resulted only in a small increase in the annexin V-positive subpopulation (Fig. 4A, compare black and grey bars). BAPTA/EGTA pretreatment completely abolished the ionomycin-induced increase in annexin V-binding (Fig 4A, compare black and white bar), confirming that the stimulation is Ca^2+^-dependent and not a non-specific effect of ionomycin addition. Microscopic examination further confirmed the pattern of labeling of the annexin V-positive parasites (Fig 4B). Taken together, these data show that scramblase activation by Ca^2+^/ionophore in parasites at the different growth phases is not sufficient to trigger binding of annexin V-FITC to the cell surface of the total parasite population.

## Discussion

One characteristic feature of the plasma membrane of eukaryotic cells is the presence of both energy-dependent and energy-independent lipid transporters that regulate the transbilayer distribution of lipids in the two monolayers. Here, we investigated the transbilayer distribution of fluorescent phospholipid analogues in the plasma membrane of *Leishmania* parasites. We found pronounced differences in the phospholipid internalization rates of different species. Species of the *L.(Leishmania)* subgenus (*L.(L.) amazonensis, L.(L.) chagasi*, *L.(L.) donovani*) rapidly internalized NBD-PC (and at a lower rate, NBD-PS), in an ATP-dependent manner even at 2°C. Because endocytosis is blocked under this condition [16], uptake of NBD-PC and NBD-PS must involve a translocation step across the plasma membrane. Species of the *L. (Viannia)* subgenus (*L. (V.) braziliensis*, *L. (V.) guyanensis)* internalized NBD-PC and NBD-PS at significantly lower rates than those observed with parasites of the *L.(Leishmania)* subgenus, indicating that the intrinsic activity of the inward translocation machinery was lower. With the exception of *L. (L.) amazonensis*, all tested species were unable to significantly transport the PE-analog NBD-PE. Previous studies on *(L.) donovani*, however, demonstrated a rapid inward redistribution of NBD-PE [19], [27]. One possible explanation for this discrepancy is that different strains were used in the two laboratories.

The differences in activity and specificity of the inward phospholipid transport correlate with the different sensitivities of the various species towards miltefosine, an alkyl phospholipid analogue of PC used in the treatment of leishmaniasis. The mechanism by which this drug is taken up is closely related to the flippase mechanism by which lipids are translocated across membranes, and these processes seem to involve the same proteins [19], [27]. How the differences in activity and specificity of the inward phospholipid transport activities relate to membrane lipid asymmetry remains to be established. Studies in the budding yeast *Saccharomyces cerevisiae* indicate that lipid asymmetry is created in the Golgi membrane *en route* to the cell membranes and that the generation of lipid asymmetry involves multiple lipid transporters, including members of the P4 subfamily of P-type ATPases [28], [29]. Similar to *S. cerevisiae*, the sequenced genomes of *L. (L.) infantum*, *L. (V.) braziliensis* and *L. (L.) major* contain multiple P-type ATPases of the P4 subfamily. A future challenge is the mapping of subcellular locations of these transporters to evaluate their transport efficiency and to establish how their activities are regulated during the life cycle of the parasite.

In addition to the ATP-dependent transbilayer transport of specific phospholipids, a previous study on *L. (L.) donovani* revealed the presence of a Ca^2+^-dependent lipid scramblase activity that can be activated by calcium ionophores [20]. In the present study, a similar scramblase activity was found in all studied species upon stimulation with the calcium ionophore ionomycin. These data suggest that the scramblase activity is a common feature in the genus *Leishmania*. Although all species had been labeled by annexin V-FITC after permeabilization with digitonin and scramblase activity could be observed in most log phase cells upon activation by the Ca^2+^ ionophore ionomycin, the binding of annexin V-FITC to the cell surface of the log-phase parasites upon scramblase activation by Ca^2+^/ionophores occurred in a lower level than that observed for the non-treated stationary phase cells.

On the other hand, stationary-phase cultures of all five *Leishmania* species contained a subpopulation of annexin V-positive parasites not present in log-phase cells. Enrichment of metacyclic forms of *L. (L.) amazonensis* displayed an even higher frequency of annexin V-positive cells, suggesting that this population contributes significantly to the increased numbers of annexin V-positive cells in stationary cultures. Notably, stimulation of metacyclics by ionomycin did only marginally increase the annexin V-positive subpopulation. Together, these results suggest that annexin V-binding lipids are confined to the cell interior of the parasites during the logarithmic growth phase of the parasite. Conceivably, when parasites enter stationary phase, these lipids are trafficked to the plasma membrane in a subpopulation of the parasites, then are translocated to and displayed on the outer leaflet of the plasma membrane. Another possibility is that the change of cell surface exposed lipids in a subpopulation of the parasites during the stationary growth phase involves head group remodeling of membrane lipids. Both possibilities would explain the lack of annexin V-binding to the majority of parasites upon ionomycin treatment despite robust scramblase activation.

Although detected in different levels, the regulated display of annexin V-binding lipids on the outer leaflet of the plasma membrane appears to be a common feature of the *Leishmania* genus and has a strikingly similarity in phylogenetically distant species of the two subgenera. Our results indicate that the observed lipid trafficking/regulation is an important feature for the parasites life cycle. It has been previously shown that pretreatment of *L. (L.) amazonensis* and *L. (V.) braziliensis* with annexin V before exposing the parasites to macrophages decreases their virulence. This suggests that annexin V is blocking a molecule with significant importance for the infection [3], [30]. Although a prime candidate, PS seems to be absent from *Leishmania* parasites [6]–[8].

Further analysis is warranted to define more precisely the phospholipid types exposed on the parasite cell surfaces and to fully uncover the proteins involved in controlling the lipid dynamics of the plasma membrane during the life cycle of *Leishmania* parasites. The characterization of proteins regulating the transbilayer lipid distribution will allow the dissection of their interactions during parasite host-cell invasion. The observed differences in the lipid translocation activities may reflect different parasite behaviors and could help define the infection course and the selection of drugs for chemotherapeutic treatment, reinforcing the importance of species identification.

## Materials and Methods

### Materials

Fluorescent 7-nitrobenz-2-oxa-1,3-diazole (NBD)-lipids, including palmitoyl-(NBD-hexanoyl)-PS (NBD-PS), palmitoyl-(NBD-hexanoyl)-PE (NBD-PE), palmitoyl -(NBD-hexanoyl)-PC (NBD-PC) and 6-NBD-hexanoyl-sphingosine-1-phosphocholine (NBD-sphingomyelin; NBD-SM), were purchased from Avanti Polar Lipids (Birmingham, AL, USA). Annexin V-fluorescein isothiocyanate (FITC) was purchased from Proteimax (São Paulo, SP, Brazil). BAPTA-AM was obtained from Molecular Probes (Life Technologies, Carlsbad, CA, USA). Miltefosine, ionomycin and all other chemicals and reagents were obtained from Sigma (Saint Louis, MO, USA) unless otherwise indicated.

### Leishmania cultures

Promastigotes of *L. (L.) amazonensis* (MHOM/BR/1973/M2269), *L. (L.) chagasi* (MCER/BR/81/M6445), *L. (L.) donovani* (MHOM/IN/80/Dd8), *L. (V.) braziliensis* (MHOM/BR/1975/M2903) and *L. (V.) guyanensis* (MHOM/BR/1975/M4147) were cultured at 25°C in M199 medium (Invitrogen, Grand Island, NY, USA) supplemented with 10% fetal bovine serum (FBS; Invitrogen) and 2% human urine [31]. Promastigote cultures were seeded at 5×10^5^ parasites ml^−1^ and harvested in log-phase(<2×10^7^ mL^−1^) or 2–4 days after reaching stationary-phase (5–7×10^7^ mL^−1^). For growth inhibition studies, log-phase promastigotes were seeded at 5×10^5^ cells mL^−1^ and treated with various concentrations (5–200 µM) of miltefosine. Cell counts were determined microscopically 48 h after the start of the incubation at 25°C. EC_50_ values refer to the concentration of miltefosine necessary to inhibit the growth rate by 50% compared to the control. Before the NBD-lipid uptake experiments, promastigotes were washed twice in HPMI (132 mM NaCl; 3.5 mM KCl; 2 mM MgCl_2_; 25 mM glucose; 20 mM HEPES pH 7.4). ATP depletion was performed by incubating the cells for 30 min at 25°C in HPMI containing 5 mM deoxyglucose (instead of glucose), 0.5 µg/mL oligomycin and 5 mM sodium azide. Stimulation with 40 µM ionomycin was performed by incubating parasites in HPMI supplemented with 2 mM CaCl_2_ for 30 min at 25°C. Calcium depletion was achieved by incubating parasites in HPMI supplemented with 100 µM BAPTA-AM (Invitrogen) and 2 mM ethylene glycol tetraacetic acid (EGTA) for 30 min at 25°C before addition of ionomycin.

### NBD-lipid uptake

NBD-lipid uptake experiments were performed essentially as described [20]. Briefly, promastigotes (2×10^7^ mL^−1^) were preincubated in HPMI supplemented with 6 µM 3-(4-octadecyl)-benzoylacrylic acid (Biomol, Hamburg, Germany) and 1 mM phenylmethanesulfonyl fluoride (PMSF) for 15 min at 25°C to block the conversion of NBD-lipids by phospholipases [16]. Unless otherwise indicated, parasites were cooled to 2°C and then labeled with 4 µM NBD-lipid. At the indicated times, 50-µL samples of the cell suspension (10^6^ parasites) were transferred to a vial containing 250 µL of HPMI with 1% (w/v) fatty acid-free bovine serum albumin, to extract NBD lipids from the cell surface. The cells were then analyzed by flow cytometry. For analysis of NBD-lipid metabolism, lipids were extracted from the cells and culture medium as previously described [32] and separated by thin-layer chromatography using a chloroform/methanol/water mixture (65∶25∶4, v/v/v). Fluorescent spots were visualized under UV light and quantified with ImageJ 1.44p (Wayne Rasband, NIH, USA).

### Annexin V-binding assay

Promastigotes (10^7^ mL^−1^) were incubated for 20 min at 10°C with 500 ng of annexin V-FITC in 0.5 ml of binding buffer (150 mM NaCl; 1 mM MgCl_2_; 1.8 mM CaCl_2_; 10 mM HEPES, pH 7.4). For plasma membrane permeabilization, digitonin was added at a final concentration of 50 µM before incubation.

### Metacyclic promastigotes enrichment

Metacyclic promastigotes from *L. (L.) amazonensis* were enriched from stationary cultures using a modified Ficoll density gradient protocol [33]. Briefly, stationary promastigotes cultures were washed in Phosphate buffered saline (PBS), ressuspended at 5×10^8^ cells mL^−1^ in 2 mL serum-free DMEM. Cells were loaded on top of a Ficoll gradient prepared in a Falcon 15 mL conical centrifuge tube and consisting of the following steps: 2 ml 20%, 2 mL 15%, and 2 mL of 10% Ficoll in serum-free M199. After centrifugation at 750 x g for 30 min, metacyclic parasites were recovered from the top of the 10% Ficoll fraction (1 ml), washed in Annexin binding-buffer (without CaCl_2_) and analyzed for annexin V-binding. Purification efficiency was assessed based on the flow cytometry methods [5], [34] and was greater than 60%.

### Flow cytometry

Flow cytometry was performed with a Becton Dickinson fluorescence-activated cell sorter (FACS, San Jose, CA, USA) equipped with an argon laser (488 nm). The data were analyzed with the Cell Quest software (BD Biosciences, San Jose, CA, USA). One microliter of 1 mg mL^−1^ PI in H_2_O was added to 200 µL of cell suspension just before analysis. Ten thousand cells were analyzed at room temperature with gating during data acquisition. Live cells were selected based on forward and side-scatter gating as well as PI exclusion. The following fluorescence channels (in log scale) were used: FL1 (530/30 nm, FITC, NBD) and FL2 (585/42 nm, PI). Data were analyzed with the Cyflogic (BD Biosciences, San Jose, CA, USA) software and the geometric mean of the fluorescence intensity was calculated.

### Microscopy

Confocal laser scanning microscopy (Zeiss LSM 510, Carl Zeiss, Oberkochen, Germany) was performed using a 100-fold magnification (a numerical aperture of 1.3) with an oil-immersion objective. FITC fluorescence was excited by an argon laser at 488 nm and then recorded between 505 and 530 nm. PI fluorescence was excited with a 559 nm HeNe1 laser and then recorded between 560 and 615 nm. Pinholes of 288 and 296 µm were used for the green and red channels, respectively.

### Data analysis

All assays were performed in triplicate and data are presented as the mean ± standard deviation (SD), unless otherwise stated. For NBD-lipid uptake experiments, data were fit to a single-exponential curve with the OriginPro 8 analysis tools (OriginLab Corporation, Northampton, MA USA) using the equation y  =  A * [1 – exp(-k * t)], where t is the time after NBD-lipid addition, A is the intracellular concentration at steady state, and k is the rate coefficient. The initial influx rate was derived from the product A*k and used consistently for presenting and comparing results. All data comparisons were tested for significance using two-tailed Student t-tests calculated with the GraphPad software; P values<0.05 were considered significant.

## Supporting Information

Figure S1 **Dot plot of FACS analysis of annexin V-FITC-binding in** ***Leishmania*** **species.** Log-phase promastigotes untreated (Log) or treated with 40 uM of ionomycin (Log/iono), and untreated stationary-phase promastigotes (Stat) were analyzed by flow cytometry after labelling for 20 min at 2°C with annexin V-FITC and propidium iodide (PI). Absolute percentages of cells are denoted in the corresponding quadrants. In the quantitative analysis of annexin V-FITC-binding, PI-positive (necrotic) cells were gated out. The percentage of annexin V-FITC-binding shown in figures 3 and 4 therefore only considers the viable cell population (PI-negative). The dot plots are representative of three independent experiments.
(TIF)
