# Supplementary figures and images for: Transbilayer Dynamics of Phospholipids in the Plasma Membrane of the Leishmania Genus
Source: PLoS One. 2013 Jan 30;8(1):e55604. doi: 10.1371/journal.pone.0055604 (PMC3559443; doi:10.1371/journal.pone.0055604)

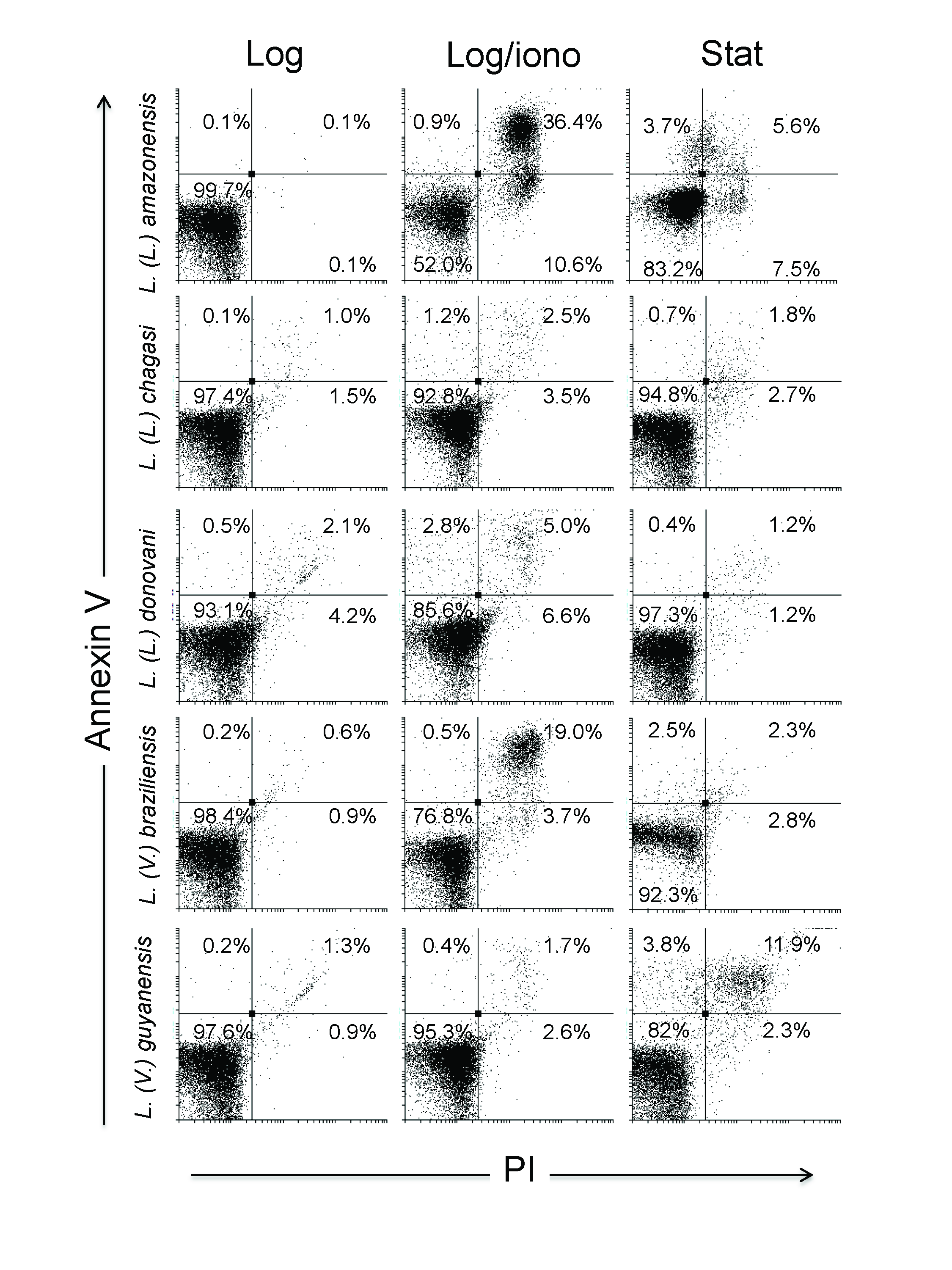

Supplement: Figure S1 — Dot plot of FACS analysis of annexin V-FITC-binding in Leishmania species. Log-phase promastigotes untreated (Log) or treated with 40 uM of ionomycin (Log/iono), and untreated stationary-phase promastigotes (Stat) were analyzed by flow cytometry after labelling for 20 min at 2°C with annexin V-FITC and propidium iodide (PI). Absolute percentages of cells are denoted in the corresponding quadrants. In the quantitative analysis of annexin V-FITC-binding, PI-positive (necrotic) cells were gated out. The percentage of annexin V-FITC-binding shown in figures 3 and 4 therefore only considers the viable cell population (PI-negative). The dot plots are representative of three independent experiments. (TIF) [file pone.0055604.s001.tif]
